# Studying the association between longitudinal nondense breast tissue measurements and the risk of breast cancer: a joint modeling approach

**DOI:** 10.1093/aje/kwae196

**Published:** 2024-07-11

**Authors:** Maya Illipse, Alessandro Gasparini, Benjamin Christoffersen, Per Hall, Kamila Czene, Keith Humphreys

**Affiliations:** Department of Medical Epidemiology and Biostatistics, Karolinska Institutet, PO Box 281, SE-171 77 Stockholm, Sweden; Department of Medical Epidemiology and Biostatistics, Karolinska Institutet, PO Box 281, SE-171 77 Stockholm, Sweden; Department of Medical Epidemiology and Biostatistics, Karolinska Institutet, PO Box 281, SE-171 77 Stockholm, Sweden; Department of Medical Epidemiology and Biostatistics, Karolinska Institutet, PO Box 281, SE-171 77 Stockholm, Sweden; Department of Oncology, Södersjukhuset, 11883 Stockholm, Sweden; Department of Medical Epidemiology and Biostatistics, Karolinska Institutet, PO Box 281, SE-171 77 Stockholm, Sweden; Department of Medical Epidemiology and Biostatistics, Karolinska Institutet, PO Box 281, SE-171 77 Stockholm, Sweden

**Keywords:** breast cancer, breast density, joint longitudinal survival modeling, time-varying covariates

## Abstract

Conflicting results have appeared in the literature on whether the amount of nondense, adipose tissue in the breast is a risk factor or a protective factor for breast cancer (BC), and biological hypotheses supporting both have been proposed. We suggest here that limitations in study design and statistical methodology could potentially explain the inconsistent results. Specifically, we exploit recent advances in methodology and software developed for the joint analysis of multiple longitudinal outcomes and time-to-event data to jointly analyze dense and nondense tissue trajectories and the risk of BC in a large Swedish screening cohort. We also perform extensive sensitivity analyses by mimicking analyses/designs of previously published studies—for example, ignoring available longitudinal data. Overall, we do not find strong evidence supporting an association between nondense tissue and the risk of incident BC. We hypothesize that (1) previous studies have not been able to isolate the effect of nondense tissue from dense tissue or adipose tissue elsewhere in the body, that (2) estimates of the effect of nondense tissue on risk are strongly sensitive to modeling assumptions, or that (3) the effect size of nondense tissue on BC risk is likely to be small/not clinically relevant.

## Introduction

A woman’s breast is a complex soft-tissue organ whose composition changes over time; it generally changes with age, especially during menopause and according to hormonal changes across the menstrual cycle. Over the last 20 years, numerous studies based on a range of populations, designs and different methods of measuring mammographic dense tissue (MD), which shows up as white or opaque on a mammogram and represents the epithelial and stromal tissue in the breast, have consistently shown that higher MD (whether considered as absolute or percent density) is associated with an increased risk of breast cancer (BC).^1^^-^^4^ The importance of MD for BC risk is discussed by Vinnicombe.^5^ One of the reasons it has attracted so much attention from breast radiologists and researchers is because of its dual importance for BC: As well as being widely accepted to be one of the strongest risk factors for BC, with a 4- to 6-fold increase in relative risk comparing women in the highest versus lowest quintiles of MD, high MD makes it difficult for radiologists to read mammograms. Research into which factors influence MD has also been plentiful.^6^^,^^7^

The relationship between mammographic breast adipose tissue and BC risk has also been researched, but there is still no consensus in the literature about whether it is a risk factor or a protective factor.^8^ Several groups of investigators have reported it to be a protective factor by demonstrating a negative association between the amount of adipose tissue in the breast, reflected by the absolute nondense area (NDA) in a mammogram, and BC risk.^4^^,^^9^^-^^11^ Stone et al^12^ found that the negative association did not persist after adjustment for MD, whereas Lokate et al^13^ found a moderate positive association (ie, reported NDA to be a risk factor). In a meta-analysis, Pettersson et al^14^ reported NDA to be a protective factor for BC, although it was not clear from that study whether this relationship was independent of dense area. In another analysis based on pooled data from 6 US studies, Bertrand et al^15^ concluded that NDA is an independent protective factor for BC. The above-mentioned publications have defined NDA in different ways; some have considered it as a categorical variable, while others have treated NDA as continuous. All studies have been cross-sectional in nature, have used standard statistical methods in their analyses, and have been based on a single baseline measurement per woman. Furthermore, not all studies adjusted for important covariates.^12^ The role of potential confounders is complex: body fat, in particular, is highly relevant to consider. Baglietto et al^16^ tried to consider the role of the adipose breast tissue in BC in the light of body fat; using an arguably ad-hoc approach, they concluded that NDA was a protective factor for BC when it was considered as a causal factor that is independent of body fat, but that when both factors were assumed to simultaneously represent adiposity, there was no association with BC.

Since the breast is a dynamic organ, researchers have suggested that longitudinal trajectories of mammographic features could be used to better understand changes in BC risk over a woman’s lifetime. It has been shown that most women show a decrease in dense tissue with age, and at the same time, they show an increase in both nondense and total breast area.^17^^,^^18^

With this article, we exploit recent advances in the methodology for joint analysis of longitudinal and time-to-event outcomes to carry out a joint analysis of trajectories of mammographic features and the risk of BC, based on data from a single large screening cohort. Moreover, we share statistical R code with which other researchers can replicate our analyses.

## Methods

### Study population

Our analysis was based on data collected from participants in a prospective, population-based Swedish screening cohort study, the Karolinska Mammography Project for Risk Prediction of Breast Cancer (KARMA).^19^ During the period from January 2011 to March 2013, women undergoing mammography screening at one of 4 hospitals in Sweden were invited to participate. A total of 70 877 women accepted, filled in an extensive web-based questionnaire, and gave blood samples. Cases of BC have been identified through a Swedish national quality register.

Both raw and digitally processed mammograms were stored, including mediolateral oblique (MLO) and craniocaudal (CC) views from full-field digital mammography systems; dense area (in cm^2^) was measured using the STRATUS method, which aligns mammograms from the same woman before taking density measurements.^20^ Here we used both views, since it has been suggested that the choice of view can affect the results of studies of nondense area^21^—the different views are 2-dimensional representations of a 3-dimensional structure, with different degrees of accuracy for estimating the area of dense and nondense portions of the breast. We extracted all processed MLO and CC images collected during screening ages (40-74 years) and constructed 2 distinct datasets. For simplicity, we only present results for the MLO dataset in the main body of the article and include results for the CC dataset in Appendix S3 of the supplementary material available online. For women eventually diagnosed with BC during follow-up, mammograms from the contralateral breast (ie, the tumor-free breast) were used, while for others, a side was randomly picked at baseline and used consistently throughout. Additional covariates on which we extracted data from KARMA were body mass index (BMI; weight [kg]/height [m]^2^), hormone replacement therapy (HRT) status, and family history of BC (FH); for the full cohort, information used to construct these covariates was collected via a questionnaire completed at study baseline. During the years 2016-2017, questionnaires were sent out to a subset of KARMA participants to collect additional information on, among other things, BMI. This information was used to perform an additional analysis, as described below. Finally, study participants were followed to a diagnosis of BC, death, or December 31, 2018—whichever came first.

### Statistical methods

**Figure 1:**
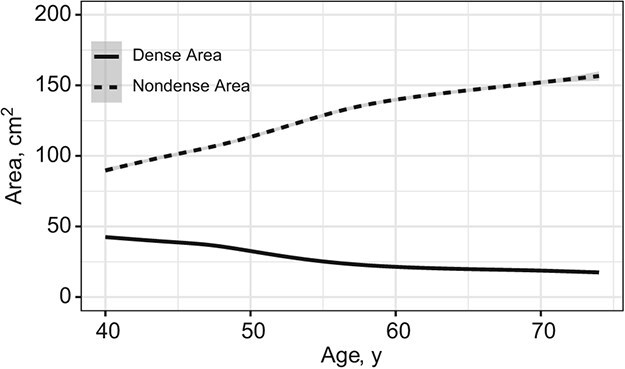
Smoothed raw trajectories for mammographic dense and nondense breast tissue areas constructed using data extracted from mediolateral oblique views, obtained using smoothing cubic regression splines on the entire study population. Data were obtained from the Karolinska Mammography Project for Risk Prediction of Breast Cancer (KARMA), Sweden, January 2011-March 2013.

A common approach to analyzing time-to-event outcomes (such as time to diagnosis of BC) is to fit a Cox proportional hazards model.^22^ This model assumes that the hazard depends only on covariates whose values are constant during follow-up (such as age at baseline, sex, or a randomized treatment). The Cox model can be extended to handle exogenous time-dependent covariates using the counting process formulation.^23^^-^^25^ While this could be considered useful in our context—trajectories of dense and nondense areas are not constant over a woman’s lifetime (Figure 1)—even this formulation is not appropriate, since our time-dependent covariates are of an endogenous nature. This approach assumes that time-dependent covariates are predictable processes measured without error, with their complete path fully prespecified. Thus, under this approach, time-dependent covariates are assumed to change value only at each follow-up visit while remaining constant during the time interval between visits. This step-function approximation is clearly unrealistic and inappropriate for mammographic features.

The joint longitudinal-survival modeling framework, which has been developing rapidly since the 1990s, can be used to properly model time-dependent, endogenous covariates together with a time-to-event outcome.^26^^-^^29^ In brief, a joint longitudinal-survival model formulates a (possibly multivariate) linear mixed-effects model for the longitudinal covariate(s), which is consequently linked to the time-to-event outcome via shared random effects. Most commonly, a single (ie, univariate) longitudinal marker has been incorporated within this modeling framework. If we used a univariate mixed-effects model for nondense area and jointly modeled the risk of BC, we could adjust for baseline dense area. However, this approach would not be sufficient for fully adjusting for the time-varying nature of dense area and its close relationship with nondense area; specifically, we could not disentangle changes in dense area over time from changes in nondense area, unless we were modeling both markers longitudinally. The recent developments in methodology and statistical software which allow for analyzing multiple markers simultaneously are therefore useful to us. Some examples of statistical software that can handle joint models with multiple markers, with different degrees of complexity, are the R packages joineRML, JMbayes2, rstanarm, VAJointSurv, and merlin^30^^-^^35^ and the Stata commands merlin and gsem.^36^^,^^37^

With the multivariate markers model, we can include both of the time-varying covariates (dense area and nondense area) together and jointly model the risk of BC. The longitudinal trajectories are then adjusted for the age at which each mammogram was taken (this is considered the time scale) and for BMI, HRT status, and family history of BC. We model the longitudinal trajectories using a natural spline with 5 degrees of freedom to allow for nonlinear effects. We do this on the square-root scale, since, if untransformed, both dense area and nondense area have skewed distributions. In order to reduce computational complexity, we assumed random intercepts only, which seemed reasonable since preliminary analyses showed negligible between-subject heterogeneity in random slopes.

For the survival submodel, we assumed a flexible, spline-based formulation for the baseline hazard (natural splines with 5 degrees of freedom on the log-hazard scale), and we adjusted for BMI, HRT status, and family history of BC. As noted above, we used age as the time scale; left-truncation of the survival outcome (ie, women had to be free of a BC diagnosis at enrollment in KARMA in order to be included in our study) was accounted for. Then, we linked the longitudinal and survival submodels using the expected value effect association structure to allow for a fair comparison with other modeling approaches.^26^

Finally, we used the limited data on BMI follow-up to fit a trivariate joint model, with an additional longitudinal submodel for BMI trajectories; in these analyses, we removed adjustment for BMI at baseline from the survival submodel, since we now included the expected value of the BMI trajectory instead. Longitudinal BMI data are presented in Figure S1 (Appendix S1). We speculate that changes in nondense area could represent a proxy for changes in BMI, thus motivating the inclusion of a third submodel to properly account for that in the analysis. The mathematical formulation of the joint models that we fit is described in more detail in Appendix S1, and we used the implementation provided by the VAJointSurv R package^34^ given that it is the only one that can account for all of the critical issues described above while still being (computationally) performant enough for the settings of our study.

Because breast tissue undergoes major changes during menopause and because body fat (and likely nondense breast tissue) has a different relationship with BC before and after menopause (García-Estévez et al^38^ showed a negative/protective association before menopause and a positive association after menopause), we fitted our bivariate and trivariate joint models to 2 subsets of the data; the first included only women who reported being postmenopausal at the start of follow-up, and the second included only women who were under age 50 years and premenopausal at the start of follow-up. We used this definition for the second group rather than defining the group to only include women who were premenopausal at baseline in order to reduce the chance of changes in menopausal status during follow-up. In addition, here in the text, we report the results of fitting the baseline-only Cox model, using both continuous nondense and dense areas and their categorization into tertiles (determined on the basis of their distribution across the entire sample), and the results of the extended Cox model analyses. The models adjusted for BMI, HRT status, family history of BC, and dense breast area. We fitted these models as they have been used in related publications, to enable comparisons. As we explained above, however, we do not consider these approaches to be particularly appropriate.

In addition to all of the above, we carried out a nested case–control study (replicating the design of Bertrand et al^15^) using KARMA data as a sensitivity analysis. This is described in more detail in Appendix S4 of the supplementary material.

All analyses for this article were performed using R, version 4.0.5,^39^ and Stata, version 17.^37^

## Results

The datasets of MLO and CC images consisted of 62 709 women (with 186 322 mammograms) and 62 851 women (with 189 801 mammograms), respectively; sample sizes differed slightly because not all views were available for all women at each screening instance. We present here results for the dataset with MLO images, with additional results shown in Appendix S2; results for the CC dataset are also included, for completeness, in Appendix S3. Note that the number of women included in each analysis (MLO or CC) was consistently the same across all methods being compared in this article, but only joint models were able to use all of the available information (including data from all follow-up mammograms beyond the first one).

Median follow-up time from inclusion in KARMA, estimated using the inverse Kaplan–Meier method,^40^ was 10.51 years (95% CI, 10.34-10.61) for the MLO data; during follow-up, 1553 women were diagnosed with BC. The median time between mammograms was 1.97 years (interquartile interval, 1.61-2.11), with a maximum of 8 mammograms per woman.

The correlation coefficient for correlation between nondense area and dense area was −0.506 (95% CI, −0.512 to −0.500). Nondense area was strongly and positively correlated with BMI, with a correlation coefficient of 0.696 (95% CI, 0.692-0.700).

Key characteristics of individuals included in each analysis dataset are described in Table 1 for MLO images only; an analogous table for CC views is presented in Table S1, alongside further information on the two subcohorts that were analyzed in this article.

**Table 1:** Baseline characteristics^a^ of persons included in a study of nondense breast tissue and breast cancer risk, by subanalysis (cohort with MLO images).^b^

**Characteristic**	**Full cohort**	**Menopausal status**	
		**Postmenopausal women**	**Premenopausal women aged <50 y**
No. of subjects	62 709	35 174	20 567
Body mass index^c^	24.46 (22.27-27.36)	24.77 (22.55-27.61)	23.88 (21.80-26.77)
Age at study entry, y	54.00 (46.00-63.00)	62.00 (57.00-67.00)	44.00 (41.00-46.00)
Family history of BC			
No	52 701 (84.0)	29 145 (82.9)	17 725 (86.2)
Yes	7882 (12.6)	4794 (13.6)	2205 (10.7)
Missing data	2126 (3.4)	1235 (3.5)	637 (3.1)
HRT status			
Never user	46 757 (74.6)	21 528 (61.2)	19 340 (94.0)
Previous user	8907 (14.2)	8172 (23.2)	396 (1.9)
Current user	2334 (3.7)	1906 (5.4)	135 (0.7)
Missing data	4711 (7.5)	3568 (10.1)	696 (3.4)
Menopausal status			
Premenopausal	25 163 (40.1)	0 (0.0)	20 567 (100.0)
Perimenopausal	2372 (3.8)	0 (0.0)	0 (0.0)
Postmenopausal	35 174 (56.1)	35 174 (100.0)	0 (0.0)
Parity			
0	7804 (12.4)	4365 (12.4)	2606 (12.7)
1	9022 (14.4)	5232 (14.9)	2878 (14.0)
2	29 652 (47.3)	16 100 (45.8)	10 327 (50.2)
≥3	15 336 (24.5)	8956 (25.5)	4564 (22.2)
Missing data	895 (1.4)	521 (1.5)	192 (0.9)
No. of mammograms			
1	6490 (10.3)	4628 (13.2)	1401 (6.8)
2	10 132 (16.2)	6541 (18.6)	2648 (12.9)
3	26 871 (42.9)	17 396 (49.5)	6787 (33.0)
4	17 391 (27.7)	6176 (17.6)	8592 (41.8)
≥5	1825 (2.9)	433 (1.2)	1139 (5.5)

Abbreviations: BC, breast cancer; HRT, hormone replacement therapy; KARMA, Karolinska Mammography Project for Risk Prediction of Breast Cancer.

^a^Values are medians (with interquartile intervals) for continuous variables and numbers (with proportions [%]) for categorical variables.

^b^Data were obtained from the KARMA study, Sweden, January 2011-March 2013.

^c^Weight (kg)/height (m)^2^.

For the full cohort, estimated hazard ratios obtained from fitting each of the models are shown in Table 2; results for the two menopausal status subcohorts are reported in Table 3. The baseline-only analysis assuming linearity (model 1) did not show a statistically significant association between nondense area and BC when using image data obtained from MLO views. This was also the case for the baseline-only analysis using nondense tissue categorized in tertiles (model 2); based on a Wald test, χ^2^ was 3.6 with 2 degrees of freedom (*P* =.17). Estimated hazard ratios for all combinations of tertiles of nondense and dense tissue area are shown in Figure S2 (Appendix S2). These plots correspond to those presented in Figure 1 of Shepherd and Kerlikowske^21^ (which in turn represent results presented in Pettersson et al^41^). Models 1 and 2 resulted in point estimates for NDA that corresponded with protective effects. For the CC data, point estimates from fitting models 1 and 2 also corresponded with protective effects, with statistical significance (at α =.05) reached in both cases (Table S2 and Figure S3 [Appendix S3]).

**Table 2:** Estimated hazard ratios for breast cancer according to mammographic nondense and dense areas from all models compared in a study of nondense breast tissue and breast cancer risk, including *P* values (MLO views only)^a^

	**Mammographic measure**			
**Model** ^**b**^	**Nondense area**		**Dense area**	
	**HR (95% CI)**	***P* value**	**HR (95% CI)**	***P* value**
Model 1	0.982 (0.956-1.009)	.190	1.147 (1.120-1.175)	<.001
Model 2				
Tertile 1	1 (Referent)		1 (Referent)	
Tertile 2	0.924 (0.808-1.057)	.248	1.572 (1.369-1.806)	<.001
Tertile 3	0.845 (0.709-1.007)	.059	2.115 (1.823-2.453)	<.001
Model 3	1.012 (0.986-1.040)	.360	1.141 (1.114-1.169)	<.001
Model 4	1.059 (1.029-1.090)	<.001	1.158 (1.130-1.186)	<.001
Model 5	1.030 (0.999-1.063)	.060	1.149 (1.121-1.177)	<.001

Abbreviations: HR, hazard ratio; KARMA, Karolinska Mammography Project for Risk Prediction of Breast Cancer; MLO, mediolateral oblique.

^a^Data were obtained from the KARMA study, Sweden, January 2011-March 2013.

^b^Model 1 is the Cox model with baseline information only and assuming continuous exposures; model 2 is the Cox model with baseline information only and categorizing dense and nondense areas in tertiles; model 3 is the Cox model with time-updated values for nondense and dense area; model 4 is the bivariate joint model for nondense and dense areas; and model 5 is the trivariate joint model including an additional longitudinal submodel for body mass index.

**Table 3:** Estimated hazard ratios for breast cancer according to mammographic nondense area from all models compared in a study of nondense breast tissue and breast cancer risk, including *P* values; analysis with MLO images only.^a^

	**Menopausal status and mammographic measure**							
	**Postmenopausal women**				**Premenopausal women aged <50 y**			
	**Nondense area**		**Dense area**		**Nondense area**		**Dense area**	
**Model** ^**b**^	**HR (95% CI)**	***P* value**	**HR (95% CI)**	***P* value**	**HR (95% CI)**	***P* value**	**HR (95% CI)**	***P* value**
Model 1	0.996 (0.964-1.029)	.810	1.139 (1.105-1.173)	<.001	(0.896-1.010)	.100	1.163 (1.102-1.227)	<.001
Model 2								
Tertile 1	1 (Referent)		1 (Referent)		1 (Referent)		1 (Referent)	
Tertile 2	1.005 (0.845-1.195)	.957	1.659 (1.425-1.932)	<.001	0.776 (0.582-1.034)	.084	1.458 (0.903-2.354)	.123
Tertile 3	0.900 (0.729-1.112)	.330	1.913 (1.606-2.278)	<.001	0.734 (0.467-1.154)	.181	2.699 (1.698-4.290)	<.001
Model 3	1.024 (0.991-1.057)	.156	1.128 (1.095-1.163)	<.001	0.987 (0.932-1.045)	.656	1.170 (1.110-1.235)	<.001
Model 4	1.052 (1.016-1.088)	.004	1.124 (1.091-1.159)	<.001	0.929 (0.869-0.994)	.032	1.172 (1.106-1.243)	<.001
Model 5	1.037 (0.999-1.076)	.053	1.120 (1.087-1.155)	<.001	0.937 (0.867-1.013)	.104	1.182 (1.117-1.252)	<.001

Abbreviations: HR, hazard ratio; KARMA, Karolinska Mammography Project for Risk Prediction of Breast Cancer; MLO, mediolateral oblique.

^a^Data were obtained from the KARMA study, Sweden, January 2011-March 2013. The table shows results from subanalyses using either (1) only women who were postmenopausal at inclusion in KARMA or (2) premenopausal women who were aged 50 years or less at inclusion.

^b^Model 1 is the Cox model with baseline information only and assuming continuous exposures; model 2 is the Cox model with baseline information only and categorizing dense and nondense areas in tertiles; model 3 is the Cox model with time-updated values for nondense and dense area; model 4 is the bivariate joint model for nondense and dense areas; and model 5 is the trivariate joint model including an additional longitudinal submodel for body mass index.

Fitting the Cox model with time-updated values of dense and nondense area (model 3) did not yield a statistically significant association between nondense area and BC, either for MLO images or for CC images.

When fitting the bivariate joint model for nondense and dense area (model 4), we found a statistically significant effect of nondense area increasing risk. An asset of the joint model is that it models association both in the true/latent levels of the markers (random effects ${b}_{i,\mathrm{ND}},{b}_{i,\mathrm{D}}$, as in the notation of Appendix S1) and in their measurement errors. The correlation between the random intercepts was estimated to be −0.63—that is, women with high DA have low NDA; the correlation between the measurement errors, ε_*i*,ND_ and ε_*i*,D_, was estimated to be $-0.43.$

When additionally modeling longitudinal trajectories of BMI (model 5), the point estimate of the effect of nondense area corresponded to a risk increase, although the evidence was weakened in comparison with model 4 and yielded a value that was no longer statistically significant. The same pattern was observed for CC images (Table S2, Appendix S3).

For the two (menopausal status) subcohort analyses, broadly speaking, the results for postmenopausal women (Table 3 and Table S3 [Appendix S3]) followed the same pattern that we observed for the full cohort. For the premenopausal women, all point estimates for effects of nondense area were below 1—that is, they indicated a protective effect, similar to the reported effect of body fat in premenopausal women.^38^ In the trivariate model the effect estimates for nondense area did not reach statistical significance.

Sensitivity analyses (reported in full in Appendix S4)—that is, analyses based on methods/designs used in other publications—also showed weak, clinically irrelevant, or even nonsignificant associations between adipose breast area and the risk of BC (Figures S4 and S5 [Appendix S4]).

Note that for the analysis based on the (where available) time-updated measures of BMI (the trivariate model), effect estimates (hazard ratios) were all closer to the null than for the bivariate joint model. It would thus be desirable to properly adjust for changes in BMI when studying the association between nondense area and the risk of BC.

Estimates of the association between NDA and BC risk were not consistent across models; however, in all analyses, dense area and BC risk were strongly associated, in the expected direction.

## Discussion

Our study, based on a single, large cohort of women undergoing mammography screening in Sweden, found no clear evidence to support the hypothesis that nondense breast area is associated with the risk of BC. The fact that estimates of the effect of nondense area are so sensitive to model assumptions (see, for instance, our comparisons between the extended Cox model and joint models) points to its effect size being small and probably not clinically relevant. Our evidence is concordant with the findings of Stone et al^12^ but at odds with those of Bertrand et al,^15^ who reported nondense area to be a protective factor following a pooled analysis of matched case–control and nested case–control studies. It is difficult to know exactly why our findings differ from those of Bertrand et al.^15^ One limitation of the analyses of Bertrand et al^15^ is that they were based on cross-sectional–only, dichotomized data on dense and nondense area, while we modeled these as continuous variables with repeated measures. Considering the dominant role of dense tissue, it is possible that conditioning on broad categories of dense area might not be sufficient for isolating the effect of nondense tissue. Moreover, the methodology used by Bertrand et al^15^ conditions on dense area, treating observed values as *true* values without accounting for measurement error; van Smeden et al^42^ showed that the impact of failing to account for measurement error can be substantial. A downside of the joint modeling approach is that, at the time of this writing, only linear association structures were allowed. More work is needed to extend the methodology and thus allowing even more flexibility.

Biologically, the role of breast fat in BC development is still not well understood. Adipose tissue derived from the breast of BC patients has shown different secretory profiles compared with those isolated from healthy individuals.^43^ Fat tissue has been described as a microenvironment promoting carcinogenesis through different mechanisms, particularly chronic inflammation.^44^^,^^45^ Breast adipose tissue secretes several growth factors that are utilized by cancer cells for their survival.^43^ In addition, adipose tissue could play a major role in BC risk, progression, migration, metastasis, and resistance to available therapies, according to Kothari et al.^43^ It has been suggested by several studies that tumor-surrounding adipose tissue can contribute to local inflammation and thus promote aggressive BC phenotypes, by its ability to secrete proinflammatory cytokines such as interleukin 6.^46^ Nevertheless, adipose tissue could potentially play a protective role. Several biological mechanisms for the negative association between NDA and BC risk have been proposed in the literature.^10^ Breast adipose tissue may store and bioactivate vitamin D, which has anticarcinogenic effects.^47^ Since the physiological atrophy of the epithelium is directly related to nondense area,^48^ breast fat could represent the level of lobular involution, which is a process inversely related to BC risk.

Mammographic density features are comprehensive measurements representing the whole breast and may have limited ability to capture local differences. Furthermore, breast adipose tissue has an important role at a microscopic level which cannot be captured at a macroscopic level (eg, via mammography). It is important to recall that MD and NDA recapitulate complex physiological and pathological conditions and do not represent a single biological state by itself. Factors that affect one of their components may also affect others, either directly or indirectly, and each component has properties that may influence the risk of BC diagnosis.^49^

We have tried to highlight some methodological difficulties in studying, from epidemiologic data, the association between nondense breast tissue and BC risk. The strong and well-established association between dense area and risk, coupled with the difficulty of obtaining accurate measurements from mammograms, is perhaps the most important factor. Use of (unsuited) statistical methods that do not take into account the time-varying nature of risk factors, and their interplay, has the potential to lead to spurious associations. We used state-of-the-art methodology and software to jointly model time-varying mammographic features and their association with the risk of BC. The methodology we used enables taking into account measurement error in mammographic features (and their correlation) extracted from images and potentially informative dropout from the study (eg, because of attrition due to death before a diagnosis of BC^50^). Nevertheless, there were limitations to our analysis. Specifically, the advanced methodology that we used requires extensive, longitudinal data on risk factors and covariates to fully and properly adjust for them in the analysis; in our case, we only had information on menopausal status at baseline inclusion in KARMA and limited follow-up data on BMI. An ideal study would regularly collect updated questionnaire data (eg, on BMI) or link to other registries and data sources, such as electronic health records.

In conclusion, we believe that epidemiologic evidence for an (at least clinically relevant) association between NDA and BC risk is questionable.

## Supplementary Material

Web_Material_kwae196

## Data Availability

R statistical code that could be used to replicate this analysis is available on GitHub at https://github.com/ellessenne/nond-bc-jm. The GitHub repository includes a simulated dataset for illustration purposes; research data from the KARMA study could be accessed by following the procedure described online at https://karmastudy.org.

## References

[1] Boyd NF , Guo H, Martin LJ, et al. Mammographic density and the risk and detection of breast cancer. *N Engl J Med*. 2007;356(3):227-236. 10.1056/NEJMoa062790 17229950

[2] Palomares MR , Machia JRB, Lehman CD, et al. Mammographic density correlation with Gail model breast cancer risk estimates and component risk factors. *Cancer Epidemiol Biomarkers Prev*. 2006;15(7):1324-1330. 10.1158/1055-9965.EPI-05-0689 16835331

[3] Vachon CM , Van Gils CH, Sellers TA, et al. Mammographic density, breast cancer risk and risk prediction. *Breast Cancer Res*. 2007;9(6):217. 10.1186/bcr1829 18190724 PMC2246184

[4] Yaghjyan L , Colditz GA, Rosner B, et al. Mammographic breast density and subsequent risk of breast cancer in postmenopausal women according to the time since the mammogram. *Cancer Epidemiol Biomarkers Prev*. 2013;22(6):1110-1117. 10.1158/1055-9965.EPI-13-0169 23603205 PMC3681889

[5] Vinnicombe SJ . Breast density: why all the fuss? *Clin Radiol*. 2018;73(4):334-357. 10.1016/j.crad.2017.11.018 29273225

[6] Vachon CM , Kuni CC, Anderson K, et al. Association of mammographically defined percent breast density with epidemiologic risk factors for breast cancer (United States). *Cancer Causes Control*. 2000;11(7):653-662. 10.1023/A:1008926607428 10977110

[7] Ursin G , Lillie EO, Lee E, et al. The relative importance of genetics and environment on mammographic density. *Cancer Epidemiol Biomarkers Prev*. 2009;18(1):102-112. 10.1158/1055-9965.epi-07-2857 19124487

[8] Velásquez García HA , Sobolev BG, Gotay CC, et al. Mammographic non-dense area and breast cancer risk in postmenopausal women: a causal inference approach in a case–control study. *Breast Cancer Res Treat*. 2018;1700(1):159-168. 10.1007/s10549-018-4737-7 29516373

[9] Torres-Mejía G , De Stavola B, Allen DS, et al. Mammographic features and subsequent risk of breast cancer: a comparison of qualitative and quantitative evaluations in the Guernsey prospective studies. *Cancer Epidemiol Biomarkers Prev*. 2005;140(5):1052-1059. 10.1158/1055-9965.EPI-04-0717 15894652

[10] Pettersson A , Tamimi RM. Breast fat and breast cancer. *Breast Cancer Res Treat*. 2012;135(1):321-323. 10.1007/s10549-012-2186-2 22855239 PMC3764603

[11] Olson JE , Sellers TA, Scott CG, et al. The influence of mammogram acquisition on the mammographic density and breast cancer association in the Mayo Mammography Health Study cohort. *Breast Cancer Res*. 2012;14(6):R147. 10.1186/bcr3357 23152984 PMC3701143

[12] Stone J , Ding J, Warren RM, et al. Using mammographic density to predict breast cancer risk: dense area or percentage dense area. *Breast Cancer Res*. 2010;120(6):R97. 10.1186/bcr2778 PMC3046440 21087468

[13] Lokate M , Peeters PHM, Peelen LM, et al. Mammographic density and breast cancer risk: the role of the fat surrounding the fibroglandular tissue. *Breast Cancer Res*. 2011;130(5):R103. 10.1186/bcr3044 PMC3262216 22030015

[14] Pettersson A , Graff RE, Ursin G, et al. Mammographic density phenotypes and risk of breast cancer: a meta-analysis. *J Natl Cancer Inst*. 2014;1060(5):dju078. 10.1093/jnci/dju078 PMC4568991 24816206

[15] Bertrand KA , Scott CG, Tamimi RM, et al. Dense and nondense mammographic area and risk of breast cancer by age and tumor characteristics. *Cancer Epidemiol Biomarkers Prev*. 2015;24(5):798-809. 10.1158/1055-9965.EPI-14-1136 25716949 PMC4417380

[16] Baglietto L , Krishnan K, Stone J, et al. Associations of mammographic dense and nondense areas and body mass index with risk of breast cancer. *Am J Epidemiol*. 2014;179(4):475-483. 10.1093/aje/kwt260 24169466

[17] Burton A , Maskarinec G, Perez-Gomez B, et al. Mammographic density and ageing: a collaborative pooled analysis of cross-sectional data from 22 countries worldwide. *PLoS Med*. 2017;14(6):e1002335. 10.1371/journal.pmed.1002335 28666001 PMC5493289

[18] McCormack VA , Perry NM, Vinnicombe SJ, et al. Changes and tracking of mammographic density in relation to Pike’s model of breast tissue aging: a UK longitudinal study. *Int J Cancer*. 2010;1270(2):452-461. 10.1002/ijc.25053 19924817

[19] Gabrielson M , Eriksson M, Hammarström M, et al. Cohort profile: the Karolinska Mammography Project for Risk Prediction of Breast Cancer (KARMA). *Int J Epidemiol*. 2017;460(6):1740-1741g. 10.1093/ije/dyw357 PMC5837703 28180256

[20] Eriksson M , Li J, Leifland K, et al. A comprehensive tool for measuring mammographic density changes over time. *Breast Cancer Res Treat*. 2018;169(2):371-379. 10.1007/s10549-018-4690-5 29392583 PMC5945741

[21] Shepherd JA , Kerlikowske K. Do fatty breasts increase or decrease breast cancer risk? *Breast Cancer Res*. 2012;14(1):102. 10.1186/bcr3081 22277587 PMC3496115

[22] Cox DR . Regression models and life-tables. *J R Stat Soc Series B Stat Methodol*. 1972;34(2):187-202. 10.1111/j.2517-6161.1972.tb00899.x

[23] Andersen PK , Gill RD. Cox’s regression model for counting processes: a large sample study. *Ann Statist*. 1982;100(4):1100-1120. 10.1214/aos/1176345976

[24] Andersen PK , Borgan Ø, Gill RD, et al. Statistical Models Based on Counting Processes. Springer Publishing Company; 1993.

[25] Fleming TR , Harrington DP. Counting Processes and Survival Analysis. John Wiley & Sons, Inc; 2005.

[26] Rizopoulos D . Joint Models for Longitudinal and Time-to-Event Data. Chapman & Hall/CRC Press; 2012.

[27] Tsiatis AA , DeGruttola V, Wulfsohn MS. Modeling the relationship of survival to longitudinal data measured with error. Applications to survival and CD4 counts in patients with AIDS. *J Am Stat Assoc*. 1995;90(429):27-37. 10.1080/01621459.1995.10476485

[28] Faucett CL , Thomas DC. Simultaneously modelling censored survival data and repeatedly measured covariates: a Gibbs sampling approach. *Stat Med*. 1996;15(15):1663-1685. 10.1002/(SICI)1097-0258(19960815)15:15<1663::AID-SIM294>3.0.CO;2-1 8858789

[29] Wulfsohn MS , Tsiatis AA. A joint model for survival and longitudinal data measured with error. *Biometrics*. 1997;53(1):330-339. 10.2307/2533118 9147598

[30] Hickey GL , Philipson P, Jorgensen A, et al. joineRML: a joint model and software package for time-to-event and multivariate longitudinal outcomes. *BMC Med Res Methodol*. 2018;18(1):50. 10.1186/s12874-018-0502-1 29879902 PMC6047371

[31] Rizopoulos D , Papageorgiou G, Miranda Afonso P. JMbayes2: Extended Joint Models for Longitudinal and Time-to-Event Data. (R package, version 0.2-0). 2022. Accessed June 3, 2023. https://CRAN.R-project.org/package=JMbayes2

[32] Goodrich B , Gabry J, Ali I, et al. rstanarm: Bayesian Applied Regression Modeling via Stan. (R package, version 2.21.3). 2022. Accessed June 3, 2023. https://CRAN.R-project.org/package=rstanarm

[33] Brilleman S , Crowther M, Moreno-Betancur M, et al. Joint longitudinal and time-to-event models via Stan. Presented at StanCon 2018, Pacific Grove, California, January 10-12, 2018. Accessed June 3, 2023. https://zenodo.org/records/1284334

[34] Christoffersen B , Clements M. VAJointSurv: Variational Approximation for Joint Survival and Marker Models. (R package, version 0.1.0). 2022. Accessed June 3, 2023. https://github.com/boennecd/VAJointSurv

[35] Martin E , Gasparini A, Crowther MJ. merlin: Mixed Effects Regression for Linear, Non-Linear and User-Defined Models. (R package, version 0.1.0). 2020. Accessed June 3, 2023. https://CRAN.R-project.org/package=merlin

[36] Crowther MJ . Merlin—a unified modeling framework for data analysis and methods development in Stata. *Stata J*. 2020;20(4):763-784. 10.1177/1536867X20976311

[37] StataCorp LLC . Stata Statistical Software: Release 17. College Station, TX: StataCorp LLC; 2021. Accessed June 3, 2023. https://www.stata.com/stata-news/news36-2/

[38] García-Estévez L , Cortés J, Pérez S, et al. Obesity and breast cancer: a paradoxical and controversial relationship influenced by menopausal status. *Front Oncol.* 2021;11:705911. 10.3389/fonc.2021.705911 34485137 PMC8414651

[39] R Core Team . R: A Language and Environment for Statistical Computing. R Foundation for Statistical Computing; 2021. https://www.R-project.org/

[40] Schemper M , Smith TL. A note on quantifying follow-up in studies of failure time. *Control Clin Trials*. 1996;17(4):343-346. 10.1016/0197-2456(96)00075-X 8889347

[41] Pettersson A , Hankinson SE, Willett WC, et al. Nondense mammographic area and risk of breast cancer. *Breast Cancer Res*. 2011;13(5):R100. 10.1186/bcr3041 22017857 PMC3262213

[42] van Smeden M , Penning de Vries BBL, Nab L, et al. Approaches to addressing missing values, measurement error, and confounding in epidemiologic studies. *J Clin Epidemiol*. 2021;131:89-100. 10.1016/j.jclinepi.2020.11.006 33176189

[43] Kothari C , Diorio C, Durocher F. The importance of breast adipose tissue in breast cancer. *Int J Mol Sci*. 2020;21(16):5760. 10.3390/ijms21165760 32796696 PMC7460846

[44] Park J , Morley TS, Kim M, et al. Obesity and cancer—mechanisms underlying tumour progression and recurrence. *Nat Rev Endocrinol*. 2014;10(8):455-465. 10.1038/nrendo.2014.94 24935119 PMC4374431

[45] Pérez-Hernández AI , Catalán V, Gómez-Ambrosi J, et al. Mechanisms linking excess adiposity and carcinogenesis promotion. *Front Endocrinol*. 2014;5:65. 10.3389/fendo.2014.00065 PMC4013474 24829560

[46] Wang YY , Lehuédé C, Laurent V, et al. Adipose tissue and breast epithelial cells: a dangerous dynamic duo in breast cancer. *Cancer Lett*. 2012;324(2):142-151. 10.1016/j.canlet.2012.05.019 22643115

[47] Ching S , Kashinkunti S, Niehaus MD, et al. Mammary adipocytes bioactivate 25-hydroxyvitamin D_3_ and signal via vitamin D_3_ receptor, modulating mammary epithelial cell growth. *J Cell Biochem*. 2011;112(11):3393-3405. 10.1002/jcb.23273 21769914 PMC3196822

[48] Ghosh K , Hartmann LC, Reynolds C, et al. Association between mammographic density and age-related lobular involution of the breast. *J Clin Oncol*. 2010;28(13):2207-2212. 10.1200/JCO.2009.23.4120 20351335 PMC2860438

[49] Boyd N , Berman H, Zhu J, et al. The origins of breast cancer associated with mammographic density: a testable biological hypothesis. *Breast Cancer Res*. 2018;200(1):17. 10.1186/s13058-018-0941-y PMC5842598 29514672

[50] Kolamunnage-Dona R , Powell C, Williamson PR. Modelling variable dropout in randomised controlled trials with longitudinal outcomes: application to the MAGNETIC study. *Trials*. 2016;17(1):222. 10.1186/s13063-016-1342-0 27125779 PMC4849065

