# Supplementary material for: Studying the association between longitudinal nondense breast tissue measurements and the risk of breast cancer: a joint modeling approach
Source: Am J Epidemiol. 2024 Jul 11;194(4):1065–71. doi: 10.1093/aje/kwae196 (PMC11978613; doi:10.1093/aje/kwae196)
Supplement: Web_Material_kwae196 [file web_material_kwae196.zip › suppl-R2.pdf]

# **Supplementary Material:** Studying the Association Between Longitudinal Non-Dense Breast Tissue and Breast Cancer Risk: A Joint Modelling Approach

## Table of Contents

**Appendix A Formulation of the Joint Longitudinal and Time to Event Models** **3**

**Appendix B Additional Results for the Mediolateral Oblique (MLO) Images Cohort** **6**

**Appendix C Results for the Craniocaudal (CC) Images Cohort** **7**

**Appendix D Sensitivity Analysis: Nested Case Control Design** **11**

## List of Figures

S1 Smoothed BMI trajectories for MLO and CC views. . . . . . . 5

S2 Hazard ratios for tertiles of dense and non-dense area and MLO views only, with 95% confidence intervals. . . . . . . . . 6

S3 Hazard ratios for tertiles of dense and non-dense area and CC views only, with 95% confidence intervals. . . . . . . . . . . . 9

S4 Hazard ratios for quartiles of non-dense breast area from the nested case-control study design and using MLO views, with 95% confidence intervals. . . . . . . . . . . . . . . . . . . . . . . . 12

S5 Hazard ratios for quartiles of non-dense breast area from the nested case-control study design and using CC views, with 95% confidence intervals. . . . . . . . . . . . . . . . . . . . . . . . 13

# List of Tables

S1 Key characteristics at baseline for individuals included in the study, by sub-analysis; cohort with CC images. . . . . . . . . . 7

S2 Estimated hazard ratios for non-dense and dense area from all models compared in this study, including p-values; CC views only. . . . . . . . . . . . . . . . . . . . . . . . . . . . . . . 8

S3 Estimated hazard ratios for non-dense area from all models compared in this study, including p-values, sub-analyses using either only post-menopausal women at inclusion in KARMA, or pre-menopausal women aged 50 years of less at inclusion. . 10

# A Formulation of the Joint Longitudinal and Time to Event Models

The univariate joint longitudinal-survival model for non-dense area and the risk of breast cancer is defined, for the $i^{th}$ subject, as

$$\begin{cases} \text{Non-Dense Area}_i = m_i(t) + \varepsilon_i(t) = X_i(t)\beta + b_i + \varepsilon_i \\ \lambda_{\text{BC},i}(t) = \lambda_0(t)\exp(W_i\gamma + \alpha g[m_i(t)]) \end{cases}$$

where $X$ represents fixed effects in the longitudinal submodel (the spline expansion of age, HRT status, BMI, and family history of breast cancer) with regression coefficients $\beta$, $b \sim N(0, \sigma^2)$ is the random intercept, and $\varepsilon \sim N(0, \sigma_\varepsilon^2)$ represents time-independent residual error. $\lambda_0(t)$ represents the baseline hazard function, $W$ the time-fixed covariates included in the survival submodel with regression coefficients $\gamma$, and $\alpha$ the association parameter quantifying the association between non-dense area and the risk of breast cancer. The function $g(\cdot)$ refers to a general association structure; in our case, we will use the expected value association structure, which is the most common parametrization for a joint longitudinal-survival model. However, `VAJointSurv` uses a different parametrization that links the deviations from the population mean of the longitudinal variables to the log-hazards of a given study subject; we denote this with $g[b_i(t)]$. More details on this parametrization can be found in the documentation of `VAJointSurv`[1]; nevertheless, it can be shown that the interpretation of the association parameter is equivalent between the two formulations.

For the bivariate joint model for dense and non-dense area, the formulation is:

$$\begin{cases} \text{Non-Dense Area}_i = m_{i,\text{ND}}(t) + \varepsilon_{i,\text{ND}}(t) = X_i(t)\beta_{\text{ND}} + b_{i,\text{ND}} + \varepsilon_{i,\text{ND}} \\ \text{Dense Area}_i = m_{i,\text{D}}(t) + \varepsilon_{i,\text{D}}(t) = X_i(t)\beta_{\text{D}} + b_{i,\text{D}} + \varepsilon_{i,\text{D}} \\ \lambda_{\text{BC},i}(t) = \lambda_0(t)\exp(W_i\gamma + \alpha_{\text{ND}} g[b_{i,\text{ND}}] + \alpha_{\text{D}} g[b_{i,\text{D}}]) \end{cases}$$

Note that in our study we included the same set of covariates $X$ in each of the longitudinal submodels and that we assume the same association structure for dense and non-dense area. $(b_{i,\mathrm{ND}}, b_{i,\mathrm{D}})$ and $(\varepsilon_{i,\mathrm{ND}}, \varepsilon_{i,\mathrm{D}})$ are both assumed to follow bivariate normal distributions.

Given that the survival submodel is a proportional hazards model, the regression coefficients $\gamma$ and the association coefficients $\alpha_{\mathrm{ND}}$ and $\alpha_{\mathrm{D}}$ can be interpreted as log-hazard ratios.

More details on the joint modeling framework and the interpretation of the association parameters can be found elsewhere[1,2].

This document includes additional results from a trivariate joint-modeling analysis as well, where we add a third sub-model for longitudinal BMI, extending the univariate and bivariate joint modeling analyses. Specifically, a second, up-to-date BMI value was collected between the end of 2016 and the beginning of 2017 by sending out questionnaires to KARMA participants. This information was available for 26 648 women included in the MLO view analysis, and for 26 750 women included in the CC view analysis. All women were included (i.e., even women with only BMI measured at baseline could contribute information). Smoothed trajectories (using cubic regression splines) for the follow-up BMI data are depicted in Figure S1, including subject-specific trajectories for a random subset of 500 KARMA participants; while, on average, changes in BMI were small, we could observe large heterogeneity between study subjects.

The trivariate joint model was formulated as:

$$\begin{cases} \text{Non-Dense Area}_i = m_{i,\mathrm{ND}}(t) + \varepsilon_{i,\mathrm{ND}}(t) = X_i(t)\beta_{\mathrm{ND}} + b_{i,\mathrm{ND}} + \varepsilon_{i,\mathrm{ND}} \\ \text{Dense Area}_i = m_{i,\mathrm{D}}(t) + \varepsilon_{i,\mathrm{D}}(t) = X_i(t)\beta_{\mathrm{D}} + b_{i,\mathrm{D}} + \varepsilon_{i,\mathrm{D}} \\ \mathrm{BMI}_i = m_{i,\mathrm{BMI}}(t) + \varepsilon_{i,\mathrm{BMI}}(t) = X_i'(t)\beta_{\mathrm{BMI}} + b_{i,\mathrm{BMI}} + \varepsilon_{i,\mathrm{BMI}} \\ \lambda_{\mathrm{BC},i}(t) = \lambda_0(t)\exp(W_i'\gamma + \alpha_{\mathrm{ND}} g[b_{i,\mathrm{ND}}] + \alpha_{\mathrm{D}} g[b_{i,\mathrm{D}}] + \alpha_{\mathrm{BMI}} g[b_{i,\mathrm{BMI}}]) \end{cases}$$

Figure S1: Smoothed BMI trajectories, for MLO and CC views. Subject-specific trajectories are also included for a subset of 500 study participants.

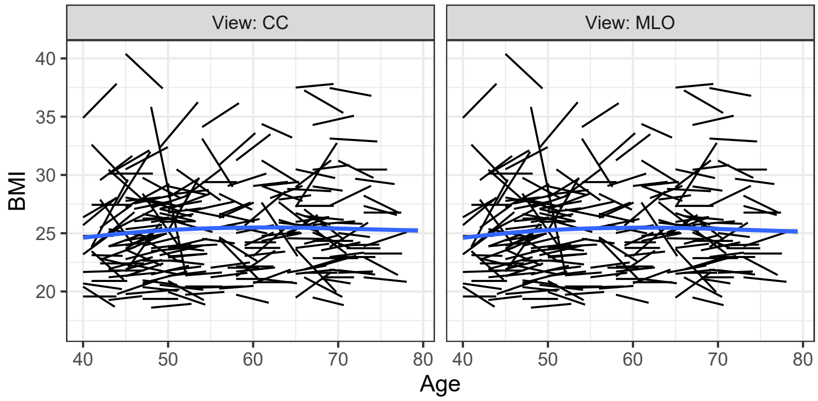


Compared to the bivariate joint model described above, $W_i'$ does not include baseline BMI (as it is now directly modeled by the longitudinal sub-model for BMI) and the sub-model for BMI only includes time as a covariate (in $X_i'$), which is modeled via a restricted cubic spline with three degrees of freedom. The longitudinal sub-model for BMI only included a random intercept term, given that only a fraction of KARMA participants returned a second questionnaire with updated BMI data. Other modeling assumptions (and included covariates) are analogous to the main analyses that were previously described.

Figure S2: Hazard ratios for each combination of tertiles of dense and non-dense area, for MLO views only, and including 95% confidence intervals. This model uses baseline data only and assumes no statistical interaction between tertiles of dense and non-dense area.

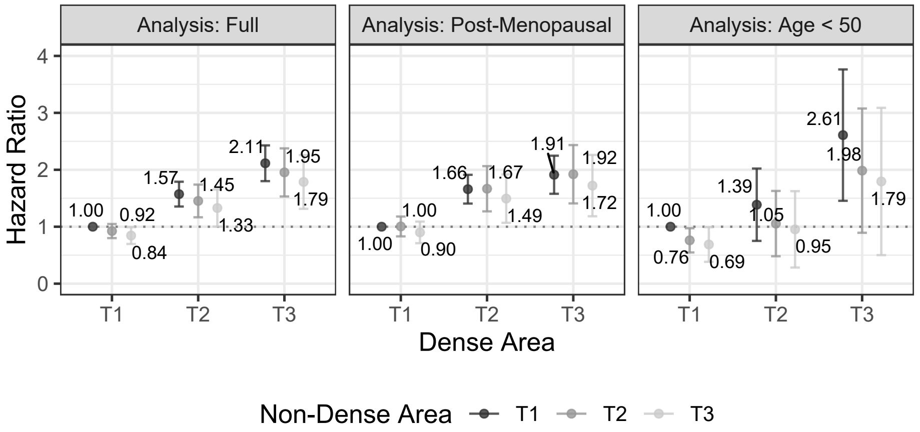


# B Additional Results for the Mediolateral Oblique (MLO) Images Cohort

Estimated hazard ratios for all combinations of tertiles of non-dense and dense tissue area are included (for MLO images only) in Figure S2. These plots correspond to those presented in Figure 1 of Shepherd and Kerlikowske[3] (which in turn represent results presented in Pettersson et al.[4]). As described in the manuscript, the effect of non-dense area was not statistically significant.

Table S1: Key characteristics at baseline for individuals included in the study, by sub-analysis; cohort with CC images. Values are medians (with interquartile intervals) for continuous variables, numbers (with proportions) for categorical variables.

| Characteristic | Full | Post-Menopausal | Pre-Menopausal, Age $<$ 50 |
|---|---|---|---|
| N. of subjects | 62,851 | 35,281 | 20,588 |
| BMI | 24.51 (22.27, 27.39) | 24.80 (22.57, 27.64) | 23.88 (21.80, 26.77) |
| Age at study entry | 54.00 (46.00, 63.00) | 62.00 (57.00, 67.00) | 44.00 (41.00, 46.00) |
| Family history of BC | | | |
| No | 52,814 (84.0%) | 29,228 (82.8%) | 17,743 (86.2%) |
| Yes | 7,900 (12.6%) | 4,808 (13.6%) | 2,207 (10.7%) |
| Missing | 2,137 (3.4%) | 1,245 (3.5%) | 638 (3.1%) |
| HRT status | | | |
| Never user | 46,847 (74.5%) | 21,585 (61.2%) | 19,360 (94.0%) |
| Previous user | 8,940 (14.2%) | 8,204 (23.3%) | 396 (1.9%) |
| Current user | 2,342 (3.7%) | 1,913 (5.4%) | 135 (0.7%) |
| Missing | 4,722 (7.5%) | 3,579 (10.1%) | 697 (3.4%) |
| Menopause status | | | |
| Pre | 25,195 (40.1%) | 0 (0.0%) | 20,588 (100.0%) |
| Peri | 2,375 (3.8%) | 0 (0.0%) | 0 (0.0%) |
| Post | 35,281 (56.1%) | 35,281 (100.0%) | 0 (0.0%) |
| Parity | | | |
| 0 | 7,823 (12.4%) | 4,376 (12.4%) | 2,610 (12.7%) |
| 1 | 9,044 (14.4%) | 5,248 (14.9%) | 2,884 (14.0%) |
| 2 | 29,700 (47.3%) | 16,138 (45.7%) | 10,332 (50.2%) |
| 3+ | 15,387 (24.5%) | 8,996 (25.5%) | 4,570 (22.2%) |
| Missing | 897 (1.4%) | 523 (1.5%) | 192 (0.9%) |
| Number of mammograms | | | |
| 1 | 5,937 (9.4%) | 4,190 (11.9%) | 1,344 (6.5%) |
| 2 | 9,235 (14.7%) | 5,917 (16.8%) | 2,463 (12.0%) |
| 3 | 27,409 (43.6%) | 18,159 (51.5%) | 6,571 (31.9%) |
| 4 | 18,428 (29.3%) | 6,593 (18.7%) | 9,053 (44.0%) |
| 5+ | 1,842 (2.9%) | 422 (1.2%) | 1,157 (5.6%) |

# C Results for the Craniocaudal (CC) Images Cohort

This section includes results for the CC cohort. Descriptive characteristics for women included in the analyses are included in Table S1; smooth raw trajectories are indistinguishable from the MLO trajectories reported in Figure 1 of the manuscript.

Median follow-up time from inclusion in KARMA, estimated using the in-

Table S2: Estimated hazard ratios for non-dense and dense area from all models compared in this study, including p-values; CC views only. Model (1) is the Cox model with baseline information only and assuming continuous exposures, model (2) is the Cox model with baseline information only and categorising dense and non-dense areas in tertiles, model (3) is the Cox model with time-updated values for non-dense and dense area, model (4) is the bivariate joint model for non-dense and dense area, and model (5) is the trivariate joint model including an additional longitudinal sub-model for BMI.

| | Non-Dense Area | | Dense Area | |
|---|---|---|---|---|
| | HR (95% C.I.) | P-Value | HR (95% C.I.) | P-Value |
| Model (1) | 0.969 (0.944-0.994) | 0.017 | 1.132 (1.106-1.160) | <0.001 |
| Model (2), Tertile 1 | Ref. | | Ref. | |
| Model (2), Tertile 2 | 0.897 (0.786-1.024) | 0.107 | 1.541 (1.344-1.766) | <0.001 |
| Model (2), Tertile 3 | 0.836 (0.706-0.990) | 0.037 | 1.977 (1.721-2.271) | <0.001 |
| Model (3) | 0.995 (0.970-1.020) | 0.679 | 1.129 (1.103-1.156) | <0.001 |
| Model (4) | 1.043 (1.015-1.071) | 0.002 | 1.142 (1.116-1.168) | <0.001 |
| Model (5) | 1.010 (0.981-1.040) | 0.494 | 1.129 (1.104-1.155) | <0.001 |

verse Kaplan-Meier method[5], was 10.52 years (95% CI: 10.34 to 10.61) for the CC data set; during follow-up, 1556 women were diagnosed with breast cancer. The median time between mammograms was the same as in the MLO data: 1.97 years, with interquartile interval (IQI) of 1.61 to 2.11.

The correlation coefficient between non-dense and dense area was $-0.379$ (95% CI: $-0.386$ to $-0.373$) for CC views, and non-dense area was strongly and positively correlated with BMI (correlation coefficient of 0.690, 95% CI: 0.686 to 0.694).

Hazard ratios for the full cohort analysis are included in Table S2. The baseline Cox model showed a significant protective effect of non-dense breast area, which was not apparent when we used more sophisticated (and arguably appropriate) statistical models. Overall, results closely matched those from the MLO analysis.

Hazard ratios by tertiles of dense and non-dense area are depicted in Figure

Figure S3: Hazard ratios for each combination of tertiles of dense and non-dense area, for CC views only, and including 95% confidence intervals. This model uses baseline data only and assumes no statistical interaction between tertiles of dense and non-dense area.

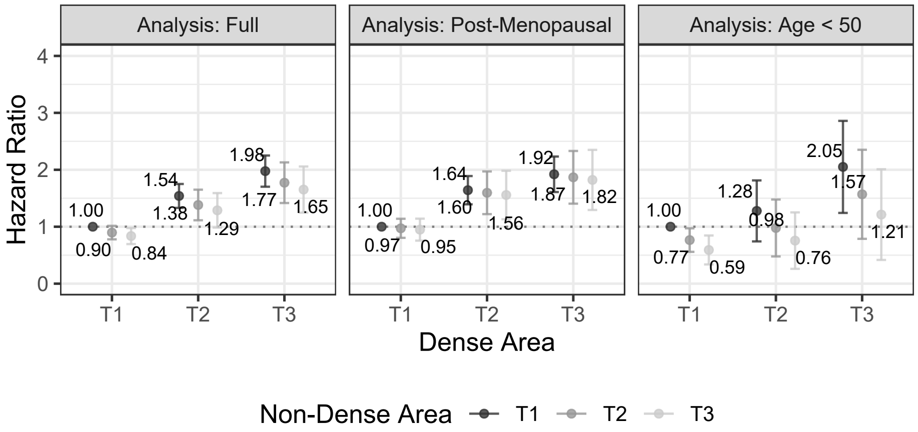


S3: as with the MLO images, the association was not statistically significant (Wald test $\chi^2 = 4.5$ with 2 degrees of freedom, p-value of 0.10).

Table S3 describes results for the sub-cohort analyses. For the post-menopausal women, there was no significant association between non-dense breast area and the risk of breast cancer. For the pre-menopausal women younger than 50 years of age, all analyses estimated a protective effect of non-dense area, although for our favored (model (5)) analysis, the effect was not statistically significant (p-value = 0.056).

Table S3: Estimated hazard ratios for non-dense area from all models compared in this study, including p-values, sub-analyses using either only post-menopausal women at inclusion in KARMA, or pre-menopausal women aged 50 years of less at inclusion. Analysis using CC images only. Model (1) is the Cox model with baseline information only and assuming continuous exposures, model (2) is the Cox model with baseline information only and categorising dense and non-dense areas in tertiles, model (3) is the Cox model with time-updated values for non-dense and dense area, model (4) is the bivariate joint model for non-dense and dense area, and model (5) is the is the trivariate joint model including an additional longitudinal sub-model for BMI.

| | Post-Menopausal Women | | | | Pre-Menopausal Women, Age $< 50$ | | | |
|---|---|---|---|---|---|---|---|---|
| | Non-Dense Area | | Dense Area | | Non-Dense Area | | Dense Area | |
| Model | HR (95% C.I.) | P-Value | HR (95% C.I.) | P-Value | HR (95% C.I.) | P-Value | HR (95% C.I.) | P-Value |
| Model (1) | 0.991 (0.961-1.022) | 0.566 | 1.128 (1.096-1.161) | <0.001 | 0.910 (0.857-0.967) | 0.002 | 1.148 (1.089-1.211) | <0.001 |
| Model (2), Tertile 1 | Ref. | | Ref. | | Ref. | | Ref. | |
| Model (2), Tertile 2 | 0.972 (0.818-1.155) | 0.748 | 1.642 (1.411-1.910) | <0.001 | 0.765 (0.584-1.003) | 0.053 | 1.278 (0.840-1.943) | 0.252 |
| Model (2), Tertile 3 | 0.948 (0.774-1.162) | 0.610 | 1.921 (1.633-2.260) | <0.001 | 0.592 (0.385-0.909) | 0.017 | 2.051 (1.383-3.041) | <0.001 |
| Model (3) | 1.015 (0.985-1.047) | 0.327 | 1.129 (1.097-1.161) | <0.001 | 0.952 (0.900-1.007) | 0.086 | 1.137 (1.079-1.198) | <0.001 |
| Model (4) | 0.999 (0.967-1.032) | 0.958 | 1.015 (0.983-1.048) | 0.358 | 0.919 (0.860-0.982) | 0.012 | 1.159 (1.097-1.226) | <0.001 |
| Model (5) | 1.020 (0.985-1.055) | 0.265 | 1.110 (1.079-1.141) | <0.001 | 0.926 (0.856-1.002) | 0.056 | 1.155 (1.091-1.223) | <0.001 |

# D Sensitivity Analysis: Nested Case Control Design

We also carried out a nested case-control study based on our cohort, for both the MLO and CC data, to mimic the analysis of Bertrand et al.[6]. We matched 5 controls per case on age, deciles of dense area, and menopause status; then, we analyzed the matched cohort using conditional logistic regression, adjusting for BMI, HRT status, family history of breast cancer, and parity. The exposure variable, non-dense breast area, was obtained from the last available mammogram (that is, the most recent value close to the event or censoring time) and it is categorized in quartiles. For this analysis, the median time between the last mammogram and the event/censoring time was 2.02 years (inter-quartile interval: 1.51 to 2.44) for the MLO data.

Results of the nested case-control analysis using the MLO data are displayed in Figure S4: we found no statistically significant association between quartiles of non-dense area and the risk of breast cancer. The Wald test statistics for the joint significance test was $\chi^2 = 1.2$ with 3 degrees of freedom, leading to a p-value of 0.76.

Repeating the nested case-control analysis using CC data, no statistically significant associations between quartiles of non-dense area and the risk of breast cancer were found once again. The Wald test statistic $\chi^2 = 0.63$, with 3 degrees of freedom, yielded a p-value of 0.89.

Figure S4: Hazard ratios (with 95% confidence intervals) for quartiles of non-dense breast area, from the conditional logistic regression analysis of a nested case-control study using data from the full cohort using MLO images.

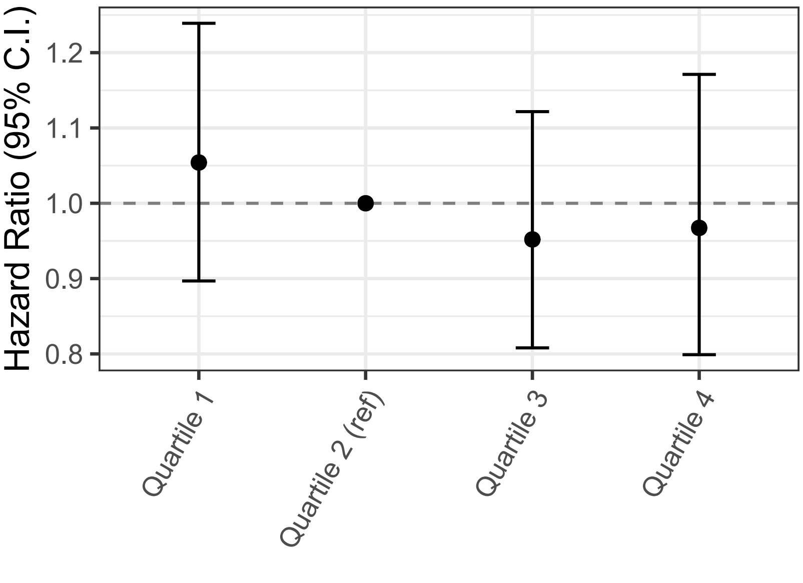

Figure S5: Hazard ratios (with 95% confidence intervals) for quartiles of non-dense breast area, from the conditional logistic regression analysis of a nested case-control study using data from the full cohort using CC images.

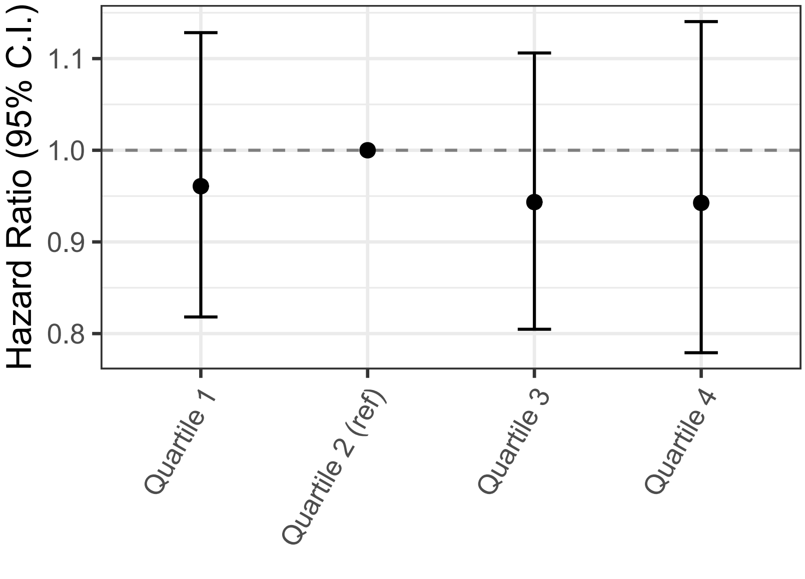

# References

[1] B Christoffersen and M Clements. *VAJointSurv: Variational Approximation for Joint Survival and Marker Models*, 2022. URL `https://github.com/boennecd/VAJointSurv`. R package version 0.1.0.

[2] D Rizopoulos. *Joint models for longitudinal and time-to-event data: With applications in R.* CRC press, 2012.

[3] JA Shepherd and K Kerlikowske. Do fatty breasts increase or decrease breast cancer risk?, 2012.

[4] A Pettersson, SE Hankinson, WC Willett, P Lagiou, D Trichopoulos, and RM Tamimi. Nondense mammographic area and risk of breast cancer. *Breast Cancer Research*, 13(5), 2011.

[5] M Schemper and TL Smith. A note on quantifying follow-up in studies of failure time. *Controlled Clinical Trials*, 17(4):343–346, 1996.

[6] KA Bertrand, CG Scott, RM Tamimi, MR Jensen, VS Pankratz, AD Norman, DW Visscher, FJ Couch, J Shepherd, YY Chen, B Fan, FF Wu, L Ma, AH Beck, SR Cummings, K Kerlikowske, and CM Vachon. Dense and nondense mammographic area and risk of breast cancer by age and tumor characteristics. *Cancer Epidemiology, Biomarkers & Prevention*, 24(5):798–809, 2015.
